# Root rot in medicinal plants: a review of extensive research progress

**DOI:** 10.3389/fpls.2024.1504370

**Published:** 2025-02-03

**Authors:** Yu Han, Tianqi Sun, Yuman Tang, Min Yang, Weiwei Gao, Lihong Wang, Chun Sui

**Affiliations:** ^1^ Institute of Medicinal Plant Development (IMPLAD), Chinese Academy of Medical Sciences & Peking Union Medical College (Key Laboratory of Bioactive Substances and Resources Utilization of Chinese Herbal Medicine, Ministry of Education & National Engineering Laboratory for Breeding of Endangered Medicinal Materials), Beijing, China; ^2^ School of Pharmacy, Heilongjiang Jiamusi University, Jiamusi, China

**Keywords:** root rot, medicinal plant, pathogen, fungi, management strategies

## Abstract

Root rot is a general term for soil-borne diseases that cause the necrosis and decay of underground plant parts. It has a wide host range and occurs in various types of plants, including crops, horticultural crops and medicinal plants. Due to the fact that medicinal plants generally have a long growth cycle and are primarily the root and rhizome herbs. This results in root rot causing more serious damage in medicinal plant cultivation than in other plants. Infected medicinal plants have shrivel or yellowed leaves, rotting rhizomes, and even death of the entire plant, resulting in a sharp decline in yield or even total crop failure, but also seriously reduce the commercial specifications and effective ingredient content of medicinal plants. The pathogens of root rot are complex and diverse, and *Fusarium* fungi have been reported as the most widespread pathogen. With the expansion of medicinal plant cultivation, root rot has occurred frequently in many medicinal plants such as Araliaceae, Fabaceae, Ranunculaceae, and Solanaceae and other medicinal plants. This article reviews recent research progress on root rot in medicinal plants, covering various aspects such as disease characteristics, occurrence, pathogen species, damage to medicinal plants, disease mechanisms, control measures, and genetic factors. The aim is to provide reference for better control of root rot of medicinal plants.

## 1 Introduction

Medicinal plants are an important component of the plant kingdom, distributed across multiple plant families and genera. They play a crucial role in ecosystems and hold significant economic and cultural value in both traditional and modern medicine. With the increasing demand for natural therapies and health products, the cultivation of medicinal plants is also on the rise. Since medicinal plants are mostly used as medicines in the form of roots or rhizomes and are grown for a long period of time, they face numerous challenges during cultivation and production. Currently, root rot disease, a typical root-related disease, poses the most severe threat to medicinal plants.

Root rot disease is a plant disease caused by various soil-borne pathogens, primarily characterized by the necrosis and decay of plant roots and root tubers (Bischoff and Goodwin, 2022). Root rot disease widely occurs in the cultivation of medicinal plants, especially in perennial root-based herbs such as *Panax ginseng* (Li Q, et al., 2022), *Panax notoginseng* (Wang P, et al., 2022), *Astragalus membranaceus* (Qi et al., 2022), and *Codonopsis pilosula* (Yu et al., 2015), severely impacting the growth of these medicinal plants. The occurrence of root rot is closely related to the growing environment, soil conditions, cultivation management practices, and continuous cropping systems of the plants cultivation site. The harm caused by root rot to medicinal plants is not only reflected in stunted growth but also directly sacrifice the yield and quality of the medicinal materials (Yang et al., 2020). When root rot occurs, the root development of effected plants is restricted, leading to diminished water and nutrient absorption capabilities, which in turn causes physiological dysfunction, wilting, yellowing, and even total plant death. Additionally, root rot hazards the accumulation of medicinal components in the plants, compromising the efficacy of the medicinal materials. Currently, the yield loss and quality decline caused by root rot has become significant bottlenecks in the development of the traditional Chinese medicine industry.

In recent years, as occurrences of root rot in medicinal plants have increased, researchers have been conducting studies on the isolation and identification of pathogens, pathogenic mechanisms, influencing factors, and control technologies. Research on pathogen types indicates that fungi from the *Fusarium* genus are among the most common pathogens responsible for root rot in medicinal plants (Zhu et al., 2023), while bacterial pathogens are also gradually recognized as proven causative factors. The damage caused by these pathogens varies note worthily among different medicinal plants. To effectively address the threat posed by root rot and enhance the production efficiency of medicinal plants, researchers continuously explore and innovate in control techniques. Currently, multiple comprehensive prevention and control strategies such as agricultural management, chemical control, and biological control have been proposed. Additionally, breeding disease-resistant varieties to mitigate root rot from a genetic perspective has also become a hot topic of interest (Chen et al., 2020).

However, despite multifarious advancements in research, the complexity and diversity of the mechanisms underlying root rot disease still presents many research challenges. This article reviews the current research status of root rot in medicinal plants, focusing on seven aspects: the typical characteristics of root rot, its occurrence, pathogens, the harm to medicinal plants, inducing factors, control strategies, and genetic factors associated with root rot. The aim is to provide a macro perspective on the current state of research on root rot in medicinal plants and to offer guidance for effective prevention and control of this disease.

## 2 Typical symptoms of root rot in medicinal plants

**Figure 1:**
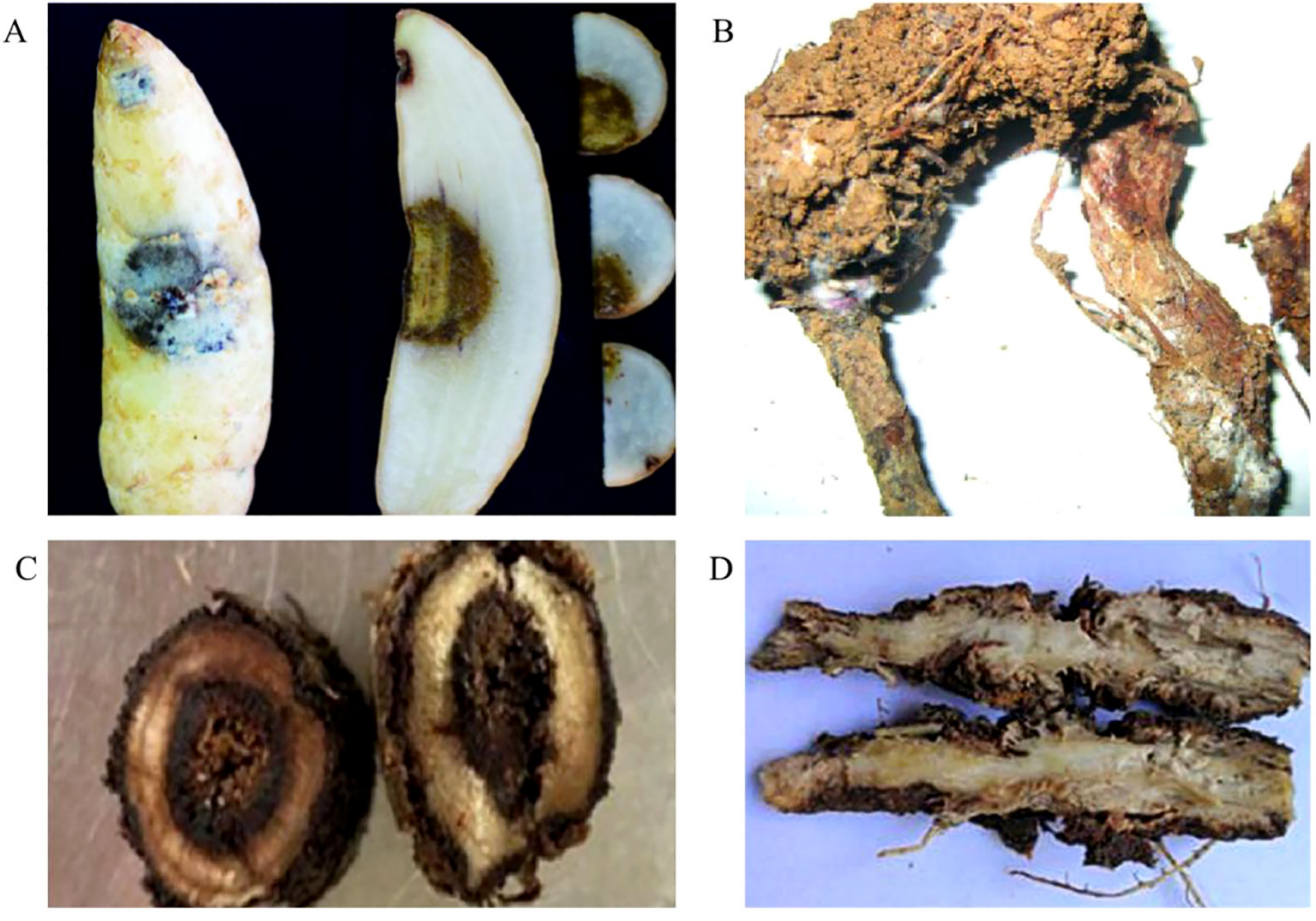
Four types of typical symptoms of root rot in medicinal plants. **(A)** The brown rot symptom on *Gastrodia elata* (Tang et al., 2022); **(B)** The dry rot symptom on *Atractylodes macrocephala* (Yang et al., 2012); **(C)** The black rot symptom on *A*. *membranaceus* (Zhang X. C. et al., 2024); **(D)** The wet rot symptoms on *Glehnia littoralis* (Lu et al., 2023).

Root rot in medicinal plants has some common typical symptoms. Usually, the progression of the disease begins with the pathogen invasion of individual branched and fibrous roots, caused leaf yellowing, branch die backs and generalized defoliation. Subsequently, it extends to the main root, causing severe damage to cells and vascular bundles. As the disease progresses, the plants struggle to absorb water and nutrients from the soil ultimately resulting in stunted growth with shrinking, yellowing and wilting leaves. In the late stage of infection, the stems of infected plant decayed with yellowed and withered leaves, and overall, culminating in the demise of the entire plants. Due to the difference of pathogens and medicinal plant species, the symptoms of root rot disease varied in some extent and often were described as wet rot, brown rot, black rot, dry rot etc. (**Figure 1**) (Li L. et al., 2021; Abbas et al., 2022).

Wet rot manifests as soft, saturated, and dark brown to black lesions on the roots, which tend to soften and may become mushy. As the condition advances, the interior of the roots progressively breaks down. This disease is frequently observed in the Araliaceae family, impacting species such as *Aralia elata*, *P. notoginseng*, and *P. quinquefolius* (Zheng et al., 2023, 2021; Zhang et al., 2020; Li et al., 2023). Plants like *Isatis indigotica* belonging to the Brassicaceae family, *A. macrocephala* from the Asteraceae family, and *Rehmannia glutinosa* from the Scrophulariaceae family are also commonly stricken by wet rot. These symptoms not only damage to the appearance of the roots but also significantly impair the plants’ ability to take up water and nutrients, leading to a decline in their health and vigor (Yang et al., 2012; Wang et al., 2013).

Brown rot affects plants, leading to wilting, necrotic lesions, and ultimately death, with the roots taking on a brown hue. This type of root rot is especially common in Ranunculaceae plants such as *Paeonia lactiflora*, *Coptis chinensis*, *P. suffruticosa*, and *Aconitum carmichaelii*, where the symptomatic brown discoloration is a key indicator (Ma et al., 2023). Moreover, brown rot is a frequent issue in the decaying parts of plants from families like Solanaceae, Asparagaceae, Orchidaceae, and Liliaceae. The disease results in the internal deterioration of the root tissues, giving them a definitive brownish tint, which can often be confused with the natural aging or drying of plant material. It is crucial to distinguish these symptoms to implement appropriate control measures (Hu et al., 2023; Liu et al., 2021).

Black rot infected plants are characterized by the emergence of black brown lesions with irregular outlines on their roots, while the leaves above ground turn yellow. In the advanced stages of the disease, the plants undergo defoliation, halt their growth, and eventually wilt and succumb. Leguminous plants, including *Medicago sativa*, *Glycyrrhiza uralensis*, and *Astragalus mongholicus*, are notably prone to this type of rot (Larsen et al., 2004; Ma et al., 2022; Guan et al., 2020). Furthermore, Camellia and members of the Gentianaceae family also have a heightened vulnerability to black rot. It is essential to monitor for these symptoms to ensure timely intervention and prevent the disease from spreading (Yang et al., 2023; Liu K, et al., 2022).

Beyond the manifestations of wet rot, brown rot, and black rot, dry rot presents its own set of characteristic symptoms. The hallmark signs of dry rot include the above ground portions of the plant becoming readily detachable, the contraction of the plant’s epidermis due to water loss, and the formation of dark brown, hollowed-out areas within the tissue. This disease is prevalent among plants in the Oleaceae family, including *Forsythia suspensa*, and also occurrences in the Taxodiaceae, Rubiaceae, Asparagaceae, and Caryophyllaceae families (Zhou et al., 2023; Zheng et al., 2022a; Liu C, et al., 2023).

## 3 Occurrence of root rot in medicinal plants

Root rot frequently affects medicinal plants that are valued for their roots, such as those in the Araliaceae, Fabaceae, Ranunculaceae, and Solanaceae families, which usually require 3-5 years of growth before they can be harvested. The incidence of this disease is alarmingly high, typically falling between 30% and 50%. In some extreme cases, the rate can exceed 70%, and there is a troubling trend of increasing severity each year (Gao et al., 2005; Li et al., 2024; Gao et al., 2014; Ma et al., 2023). Therefore, the establishment of effective prevention and treatment measures for root rot is significantly more critical for these perennial medicinal plants than for annual crops.

### 3.1 The occurrence of root rot in China

In China, the root rot was initially identified in 1989, that subsequent reports indicating its presence in northwest (Xinjiang, Gansu) and northeast (Heilongjiang, Jilin). (Zhang J. et al., 2024; Guan et al., 2020a; Liu et al., 2022; Li et al., 2022; Bernardi-Wenzel et al., 2016). These regions are significant pastoral and agricultural hubs (**Table 1**) (Wu W. et al., 2020; Yang Y. et al., 2024; Zhang Z. et al., 2021; Ni et al., 2021; Sun X. et al., 2024; Shen et al., 2022; Tang et al., 2020; Wu H. et al., 2020; Su and Fu et al., 2013; Cai F. et al., 2021; Chu et al., 2024; Li and Cheng, 2021). Notably, root rot of leguminous plants often occurs in Heilongjing, Liaoning, and Tibet (Guan et al., 2020a; Abbas et al., 2022). For plants in the Araliaceae family, root rot is commonly reported in Jilin and Yunnan (Zhang et al., 2020; Gao et al., 2014; Mao et al., 2014). Gansu has emerged as a region where root rot is prevalent among plants from the Apiaceae family, Platycodon family, and legumes family (Zhang J. N., et al., 2024; Zhao et al., 2021; Cai W, et al., 2021), while in Guizhou and Sichuan, the disease also occurs frequently (Wu H. et al., 2020; Chen et al., 2020). It can be seen that root rot has caused extensive damage to the production of medicinal plants in China (Li et al., 2020; Liu et al., 2023; Zhu et al., 2020; Zheng et al., 2022b; Miao et al., 2006; Liu N. et al., 2020; Wang H. et al., 2024; Zhang C. et al., 2021; Wu X. et al., 2020).

**Table 1:** The climate zone distribution in China where root rot disease has been reported occurring in medicinal plants.

Medicinal plant	Fungi	Climate zone	Latitude and longitude	Incidence rate
*R. glutinosa*	*F. solani* ^1^	Temperate continent	34°79′N, 113°79′E	*-*
*A. sinensis*	*-*	Temperate continent	34°53′-35°25′N, 103°44′-104°20′E	*-*
*P. notoginseng*	*-*	Tropical monsoon	25°33′98′′N, 103°03′72′′E	*-*
*C. chinensis*	*-*	Subtropical monsoon	30°01′99′′N, 106°26′48′′E	*-*
*P. ginseng*	*-*	Temperate monsoons	41°42′-42°25′N, 127°28′-128°16′E	*-*
*G. elata*	*-*	Subtropical monsoons	28°16′-28°22′N, 107°55′-108°22′E	25%
*C. pilosula*	*-*	Temperate monsoons	34°53′-35°25′N, 103°44′-104°20′E	*-*
*M. sativa*	*-*	Temperate continent	38°21′30′′-39°00′30′′N, 101°34′41′′-102°34′26′′E	*-*
*L. chuanxiong*	*-*	Subtropical monsoon	31°08′11′′N, 104°02′14′′E	*-*
*R. glutinosa*	*F. oxysporum* ^2^	Subtropical monsoon	34°52′-35°2′N, 112°51′-113°13′E	*-*
*Pulsatilla koreana*	*-*	Temperate continent	38°43′-43°26′N, 118°53′-125°46′E	45%
*C. pilosula*	*-*	Temperate continent	34°53′-35°25′N, 103°44′-104°20′E	20%
*A. sinensis*	*-*	Temperate monsoon	34°53′-35°25′N, 103°44′-104°20′E	*-*
*P. notoginseng*	*-*	Tropical monsoons	25°33′98′′N, 103°03′72′′E	*-*
*C. chinensis*	*-*	Temperate monsoons	30°01′99′′N, 106°26′48′′E	*-*
*P. ginseng*	*-*	Temperate monsoons	41°42′-42°25′N, 127°28′-128°16′E	*-*
*M. sativa*	*-*	Temperate continent	38°21′30′′-39°00′30′′N, 101°34′41′′-102°34′26′′E	*-*
*P. odoratum*	*-*	Temperate monsoons	41°23′32′′N, 124°04′27′′E	40%-50%
*Gentiana scabra bunge*	*-*	Temperate monsoons	41°47′28′′N, 124°21′35′′E	25%
*L. chuanxiong*	*-*	Subtropical monsoon	30°54′-31°26′N, 103°40′-104°10′E	*-*
*A. sinensis*	*F. redolens* ^3^	Temperate continent	35°00′39′′N, 104°63′49′′E	*-*
*M. sativa*	*-*	Temperate continent	38°21′30′′-39°00′30′′N, 101°34′41′′-102°34′26′′E	*-*
*A. sinensis*	*F. avenaceum* ^4^	Temperate continent	34°26′22′′N, 104°02′13′′E	*-*
*C. pilosula*	*-*	Subtropical monsoon	34°53′-35°25′N, 103°44′-104°20′E	*-*
*C. chinensis*	*F. tricinctum* ^5^	Temperate monsoons	30°01′99′′N, 106°26′48′′E	*-*
*Ophiopogon japonicus*	*F. acuminatum* ^6^	Subtropical monsoons	30°83′N, 112°53′E	75%
*M. sativa*	*F. verticillioides* ^7^	Plateau continent	38°21′30′′-39°00′30′′N, 101°34′41′′-102°34′26′′E	*-*
*M. sativa*	*F. proliferatum* ^8^	Temperate continent	38°21′30′′-39°00′30′′N, 101°34′41′′-102°34′26′′E	*-*
*Schisandra chinensis*	*-*	Temperate monsoons	21°29′57′′N, 122°52′33′′E	
*A. carmichaelii*	*-*	Subtropical monsoon	31°47′N, 104°45′E	
*M. sativa*	*F. equiseti* ^9^	Temperate continent	38°21′30′′-39°00′30′′N, 101°34′41′′-102°34′26′′E	*-*
*R. glutinosa*	*F. moniliforme* ^10^	Subtropical monsoon	34°52′-35°2′N, 112°51′-113°13′E	*-*
*M. sativa*	*F. thapsinum* ^11^	Plateau continent	31°90′′-39°19′N, 89°35′-103°04′E	*-*
*M. sativa*	*F. incarnatum* ^12^	Temperate monsoons	44°04′-46°40′N, 125°42′-130°10′E	*-*
*P. quinquefolius*	*F. proliferatum* ^13^	Temperate monsoons	33°17′42′′-33°53′29′′N, 106°38′05′′-107°18′14′′E	*-*
*Dendrobium officinale*	*F. sambucium* ^14^	Subtropical monsoon	28°31′N, 119°27′E	*-*
*M. sativa*	*T. roseum* ^15^	Plateau continent	31°90′′-39°19′N, 89°35′-103°04′E	*-*
*C. pilosula*	*I. robusta* ^16^	Temperate continent	30°45′59′′N, 109°36′36′′E	*-*
*P. quinquefolius*	*C. destructans* ^17^	Tropical monsoons	33°17′42′′-33°53′29′′N, 106°38′05′′-107°18′14′′E	27.4%
*R. glutinosa*	*A. niger* ^18^	Subtropical monsoon	34°52′-35°2′N, 112°51′-113°13′E	*-*
*R. glutinosa*	*P. cactorum* ^19^	Subtropical monsoon	34°52′-35°2′N, 112°51′-113°13′E	*-*
*R. glutinosa*	*T. basicola* ^20^	Subtropical monsoon	34°52′-35°2′N, 112°51′-113°13′E	*-*
*L. chuanxiong*	*Ph. glomerata* ^21^	Subtropical monsoon	30°54′-31°26′N, 103°40′-104°10′E	*-*
*L. chuanxiong*	*P. cucumerina* ^22^	Subtropical monsoon	30°44′54′′-31°22′09′′N, 103°25′42′′-103°47′E	*-*
*P. notoginseng*	*A. tenuis* ^23^	Tropical monsoons	23°16′-23°44′N, 103°43′-104°27′E	*-*
*P. notoginseng*	*Pseudomonas* sp.^24^	Tropical monsoons	25°33′98′′N, 103°03′72′′E	*-*
*A. sinensis*	*P. syringae* ^25^	Temperate monsoon	34°53′-35°25′N, 103°44′-104°20′E	*-*
*P. notoginseng*	*C. indoloqenes* ^26^	Tropical monsoons	23°16′-23°44′N, 103°43′-104°27′E	*-*

**Figure 2:**
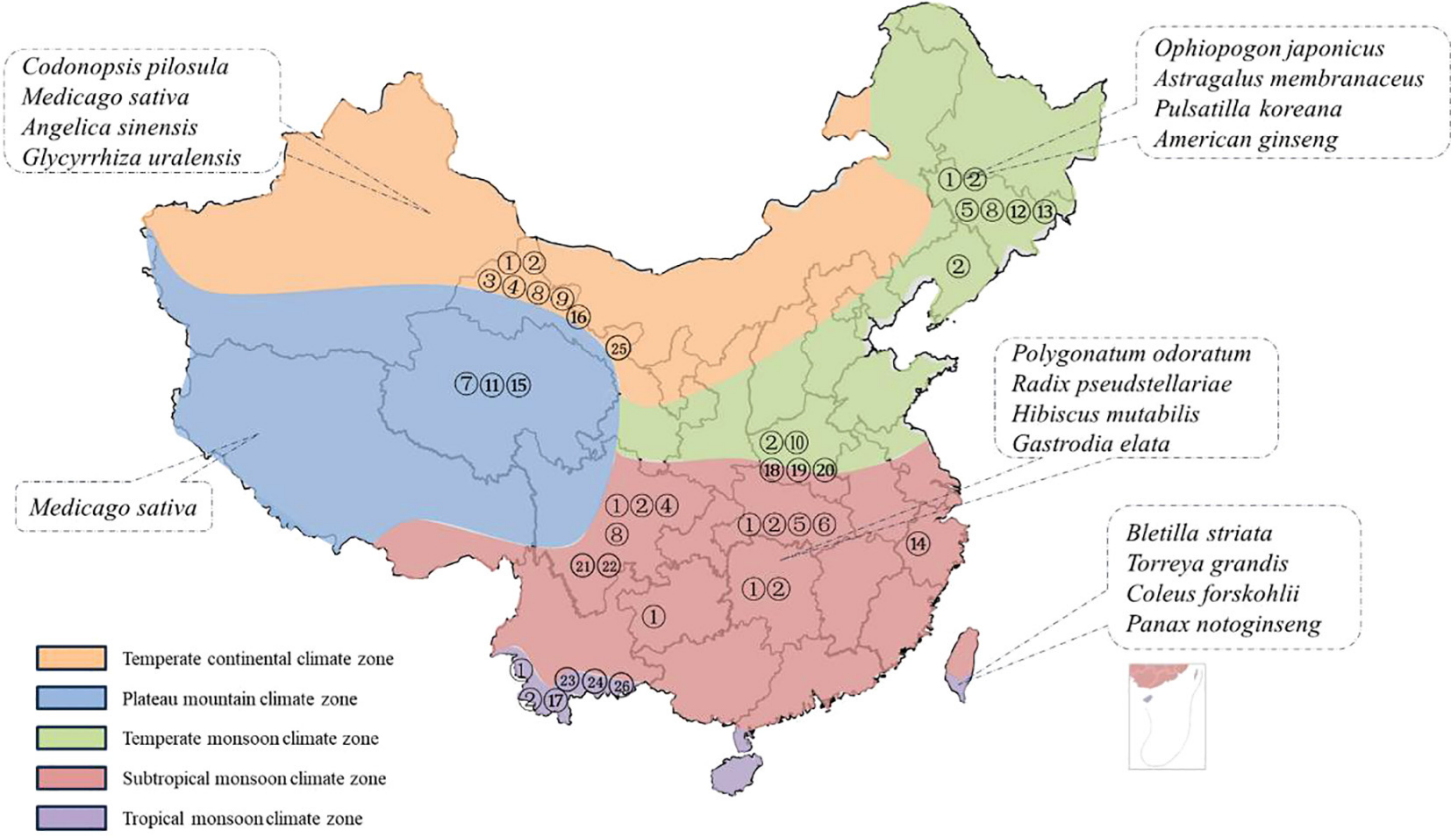
Medicinal plants susceptible to root rot in different climatic zones of China. (The medicinal plants listed are the species with the highest morbidity in each climatic condition. The numbers correspond to the pathogen numbered in **Table 1**).

* The “-” in the column of fungi represents the pathogen above. The “-” in the column of incidence rate represents no data. The superscript numbers in the column of fungi correspond to the numbers in **Figure 2**, indicating the same pathogen.

The soil texture can significantly impact the distribution of pathogens within the soil as well as the plants resistance to diseases (Cruz et al., 2020; Nielsen et al., 2009; Wang Y. et al., 2023; Wang Y. et al., 2022). The International Soil Texture Classification System categorizes soil textures into four major groups based on the content of clay particles: sandy soils, loam soils, clay loam soils, and clay soils. Each of these soil textures exhibits distinct properties. According to the current literature reports, the soil type in most root rot disease areas is loam soil, which can be seen to cause the incidence of root rot disease (Zhang et al., 2020; Gao et al., 2014; Zhang et al., 2024; Mao et al., 2014). Beyond soil factors, climatic conditions exert a more substantial influence on the occurrence of root rot disease. Considering the diverse climatic conditions across the country, China is categorized into five climatic regions: tropical, subtropical, temperate, cold, and plateau. In the subsequent discussion, we will delve into the classification based on the climatic divisions of production areas and the corresponding distribution of root rot in medicinal plants (**Figure 2**).

### 3.2 Disease onset status in other countries

As a global soil disease, root rot has caused damage to medicinal plants in various countries such as the United States, India, Vietnam, and Hungary (Liyanapathiranage et al., 2023; Moparthi et al., 2021). Root rot of the medicinal plant *Gynostemma pentaphyllum* has been discovered and studied at Tam Dao, Vinh Phuc, Vietnam (Chu et al., 2022). Nagy et al. discovered symptoms of root rot in common sage in Somogy and Zala counties in Hungary (Nagy et al., 2024). Root rot shows various symptoms like yellowing and wilting of leaves, brown to black roots, oozing, putrefaction and decaying of roots (Hu et al., 2021; ÖLmez et al., 2021). The distribution of medicinal plant root rot is intricately linked to environmental factors and soil types. According to foreign research, the occurrence of root rot is also closely related to soil types. The soil types of root rot endemic areas in Hungary, southern India, Vietnam and the United States are loamy. This conclusion is consistent with the results of soil types in China (Li et al., 2023). In Hungary, *Salvia officinalis* is often infected by *Phytophthora pseudocryptogea*. The disease occurred at a frequency of 15-20% (Nagy et al., 2024). Root rot is the major disease of *C. forskohlii* causing heavy losses (>50%) in South India (Singh et al., 2011). In areas with limited rainfall, like certain regions in the United States and Australia, plant thrives, particularly during rainless years. On the other hand, regions experiencing high rainfall, such as eastern Australia and cooler elevated areas in the United States, witness the dominance of climate change. Indian and North Dakota root rot is also found on *Nepeta cataria*, *Tagetes erecta*, and *Cannabis sativa*, which are often damaged by *F. solani*, with an incidence rate of up to 30% (Nishad et al., 2018; Saroj et al., 2013; Khan et al., 2022).

## 4 Root rot causing pathogens

Root rot in medicinal plants is primarily caused by pathogenic fungus, while bacteria and nematodes can also cause disease (Guan et al., 2020b). However, little research has been conducted on these non-fungal pathogens recently. There are numerous pathogenic fungi, each with unique structural characteristics and pathogenic symptoms. As a result, a thorough understanding for the characteristics of these fungal diseases is crucial for the successful control of root rot in medicinal plants, both theoretically and practically value. The correct identification of pathogenic fungi provides a scientific foundation for the development of targeted control measures to protect the healthy growth of medicinal plants and ensure the quality and production of medicinal herbs.

Among the known fungal pathogens, *Fusarium* is widely recognized as a significant pathogenic bacterium for medicinal plant root rot. This fungus may live in soil for long periods of time and is spread by rain or irrigation water. *F. solani* and *F. oxysporum* are the most common pathogens responsible for root rot in a wide range of medicinal plants, including *A. sinensis*, *P. ginseng*, *P. notoginsen*g, *C. chinensis* and *G. uralensis* (De Lamo and Takken, 2020; Coleman, 2016; Cao X. 2013; Liang et al., 2022). In addition, *F. redolens*, *F. avenaceum* and *F. tricinctum* have also been documented to cause the condition (Wang Y. et al., 2022; Cao X. 2013, Liang et al., 2022). The genus of *Fusarium*, being the most common causative agents of root rot, have been extensively studied and widely focused on. Common modes of transmission for *Fusarium* include vertical and horizontal spread (Nelson, 1992). The former involves transmission to the next generation of seeds or asexual propagation through infected plant breeding. The latter occurs via mycelium lurking in the soil and spores in the air, which infect susceptible plant tissues (Headrick and Pataky, 1991; Wang L, et al., 2024). The genus *Fusarium* interacts with its host after infection, resulting in physiological interactions or the production of secondary metabolites that trigger the development of root rot in plants (Lin et al., 2014). Wang et al. found that after inoculation with *M. sativa*, the content of soluble sugars, soluble proteins, and malondialdehyde significantly changed in multiple alfalfa varieties (Wang S, et al., 2023). Haidoulis et al. performed transcriptome analysis on the roots of small-grain cereals at the early stages of *F. graminearum* infection, revealing that the expression of core tissue-specific genes, such as those for cell wall-degrading enzyme synthesis, was elevated. Additionally, some tissue-dependent genes, including those for aurofusarin production and cutin degradation, also showed increased expression (Haidoulis and Nicholson, 2022). *F. solani* also changes the expression of alkaloid-related bio-synthetic genes in the roots of *C. chinensis* thereby reducing the synthesis of active medicinal components (Song X, et al., 2023). The genus *Fusarium* produces many types of toxins, leading to diverse symptoms and levels of damage in plants, with fusaric acid (FA) being the primary one. FA can cause programmed cell death in various plant species. Reveglia et al. revealed how plant toxins from *F. acuminatum* (such as FA) and other potential metabolites cause wilting in grapevine plants (Reveglia et al., 2018).

The occurrence of root rot in medicinal plants, apart from being induced by pathogenic *Fusarium* species, is also influenced by the co-action of other pathogens. For example, the root rot pathogens of *Angelica sinensis* include *F. solani*, *F. oxysporum*, *F. acuminatum*, *F. redolens*, *F. avenaceum* and *R. solani*. Of these, *F. acuminatum* is the main causal agent (Zhang T. et al., 2024). The root rot pathogens of *R. glutinosa* include *F. solani*, *F. oxysporum*, *A. niger*, *T. basicola* and *R. solani*. The main causal agent is the fungus *Fusarium* sp (Wu et al., 2021). The same fungus, however, can cause root rot in a variety of medicinal herbs. For example, the most common *F. solani* not only infests *A. sinensis*, but may also infest herbs *P. notoginsen*g, *C. chinensis*, *P. ginseng* and *C. tangshen* (Farh et al., 2018).

Some bacteria are also capable of causing root rot in medicinal plants, but there is limited literature on this. *Pseudomonas* sp. and *C. indoloqenes* can cause root rot in *P. notoginseng*. Similarly, *P. syringae* can cause root rot in *A. sinensis* (Zhang M. et al., 2021). In addition, when the roots of medicinal plants are damaged by underground pests and nematodes, these wounds facilitate the invasion of pathogens that accelerate and exacerbate the development of root rot. Therefore, bacterial, subterranean pest and nematode infestations also play an important role in the development of root rot and need to be taken into account during disease management.

**Figure 3:**
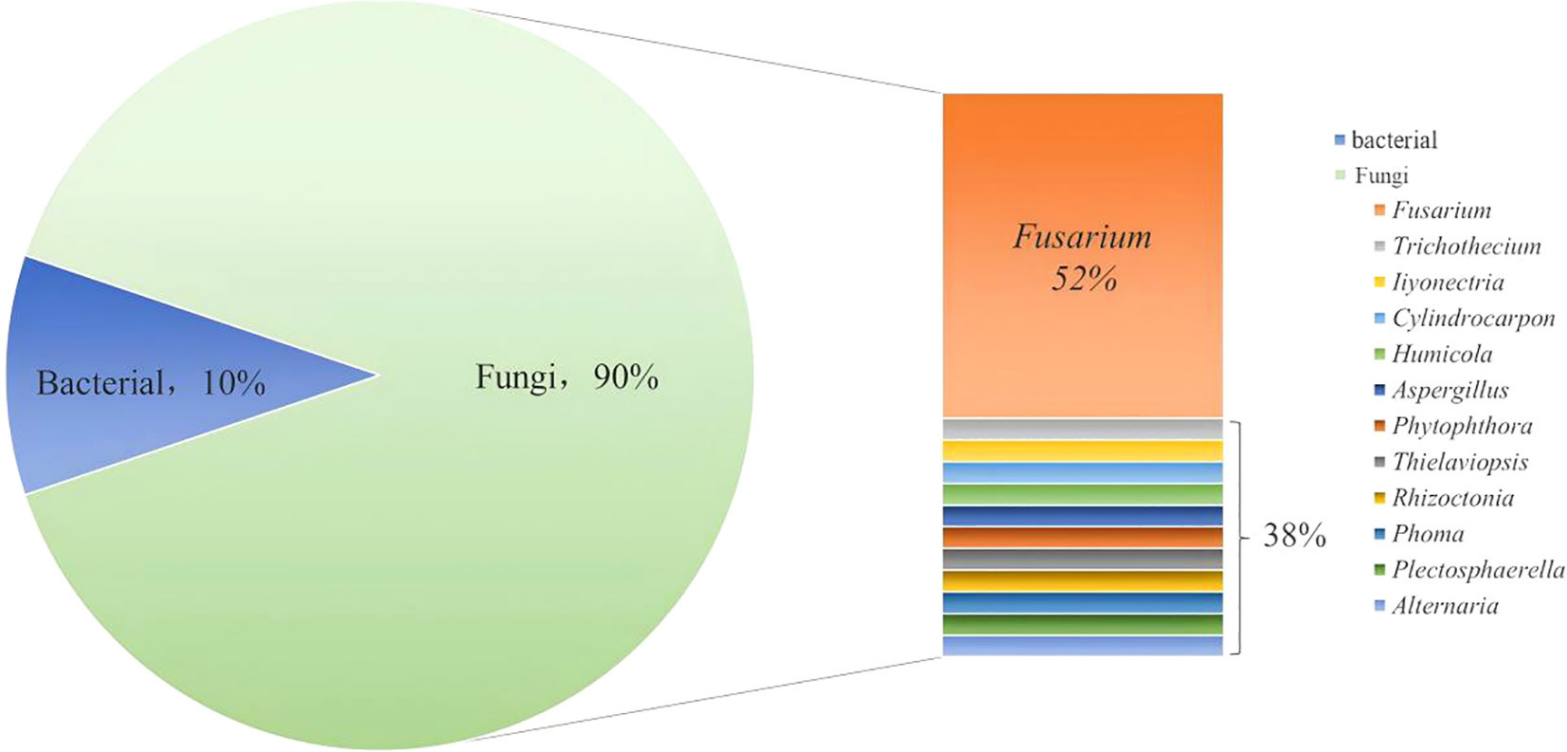
Pathogens of root rot disease in medicinal plant. (Statistics by type of pathogens).

Different medicinal plants may be affected by pathogens that are peculiar to them. Therefore, exploring the pathogen of root rot in different medicinal plant is critical for developing a precise control approach to prevent and manage the root rot diseases. The pathogens that have been reported in the literature so far are summarized in **Figure 3**.

## 5 The damage of root rot to medicinal plants

Root rot has extensive and profound implications for medicinal plants, resulting in decreased production and quality. The disease attacks the plant’s roots, causing root rot, which decreases the plant’s ability to absorb water and nutrients. As the disease advances, the plant’s growth and development are inhibited, perhaps leading to plant mortality. This not only reduces the availability of medicinal plants, but also has a substantial impact on the economics of their production. The damage of root rot on medicinal plants in terms of production and quality will be discussed below.

### 5.1 Decrease in production

Root rot poses a serious threat to the production of medicinal plants and is capable of triggering varying degrees of production reduction. In the case of *Salvia miltiorrhiza*, a severe incidence of root rot may result in a production reduction of more than 50%. The root rot of *Beta vulgaris* was initially discovered in the United States and has since emerged in countries such as India and China. The disease is particularly severe in the Chinese provinces of Heilongjiang and Jilin, where it typically causes a 10%-40% reduction in *B. vulgaris* production, and in severe cases may even lead to complete crop failure. In addition, *M. sativa* has been severely infected by root rot in several countries around the world, including the United States, Canada, Australia, Russia, Japan and Argentina. The disease has also been recorded in Xinjiang, Qinghai and Gansu regions of China. Root rot can cause mortality rates in *M. sativa* ranging from 60.08% to 73.45%, resulting in annual production reductions of about 20% globally. In some severe cases, the reduction in production can be as high as 40% (Hu, 2009; Chatterton, 2022). The problem of root rot disease during cultivation of *P. quinquefolium* is also prominent, with a perennial incidence of about 30%, and up to 90% in severe cases (Sun et al., 2023). Root rot of *R. glutinosa* indicus generally leads to a production loss of 10% ~ 30%, and in severe cases, it reaches more than 50% (Wang et al., 2013). *P. notoginseng* suffers annual losses of 5% ~ 20% due to root rot, and in severe cases, it can reach 70% or even crop failure (Liao et al., 2006). All these data emphasize the profound impact of root rot on the yield and agricultural production of medicinal plants.

### 5.2 Decline in quality

Root rot poses a serious threat to the quality of medicinal plants while reducing their production. This disease not only causes diseased spot on the medicinal parts, directly reducing the commercial value, but also reduces the content of important active components in medicinal plants, thereby lowering the overall quality of the herbs. In addition, the accumulation of toxins produced by root rot pathogenic bacteria can exacerbate root rot and in severe cases lead to the death of the entire plant. These factors work together to affect the commercial and medicinal value of medicinal plants.

The quality of Chinese medicinal materials is usually evaluated by their appearance characteristics. Medicinal plants that are seriously affected by root rot, such as *L. chuanxiong*, *P. quinquefolium* and *P. ginseng*, have brown or black spots on their roots (Sun X, et al., 2024; Lan et al., 2022; Farh et al., 2018). These diseased spots greatly affect the appearance and quality of the medicinal herbs, resulting in lower market prices for them. In addition to affecting the appearance of herbs, root rot can damage the structure and function of the roots of medicinal plants, preventing the synthesis and accumulation of active components in the roots from being impeded. Studies show that the degree of decay of *P. quinquefolium* root is closely related to its saponin content. Specifically, the content of the two monomeric saponins, Re and Rb_1_, is significantly reduced in the more severely decayed roots of *P. quinquefolium* (Fan et al., 2021). Rahman’s study discovered that the amount of six key monomeric saponins (Re, Rg_1_, Rb_1_, Rb_2_, Rc and Rd) is reduced by 40 to 50% in diseased *P. quinquefolium* root (Rahman and Punja, 2005). Ginsenosides have the properties of strengthening the body, regulating the nervous system and slowing down the aging process. When *P. ginseng* is attacked by root rot, the content of ginsenosides will also be significantly reduced (Guan et al., 2020; Bischoff and Goodwin, 2022). Triterpenoid saponins and flavonoids in *A. membranaceus* roots, compounds that are important in immune enhancement and anti-inflammatory properties, are likewise reduced in content by the effects of root rot (Qi et al., 2022). Furthermore, after invading the roots of medicinal plants, the pathogenic bacteria will continuously produce conidia and toxins. The accumulation of these toxins exacerbates the degree of root infestation and seriously affects the plant’s normal absorption and utilization of soil nutrients and water, which in turn leads to yellowing and wilting of the above-ground portion of the plant and reduces the quality of the medicinal plant (Xu et al., 2021). For example, fungi of the genus *Fusarium* produce fusaric acid, a non-specific toxin that can reduce root vigor by altering the permeability of the plant’s root membrane (Hong et al., 2023). *R. solani* toxin can damage cell structure, causing disintegration or even disappearance of cell membranes, as well as leading to changes in organelles and nucleus structure (Qin et al., 2020). Studies shows that the *Rhizoctonia solani* toxin also induces the synthesis of chitinase and β-1, 3-glucanase in rice plants (Sriram et al., 2001). Mycotoxins produced by *Aspergillus niger*, Monocephalosporin compounds in Trichothecene mycotoxins and toxins produced by P. coctarum are also important causes of root rot in medicinal plants (Jin et al., 2015, 2007). Other mycotoxins causing root rot have not been reported.

Root rot has a significant impact on medicinal plants, causing a partial reduction in plant productivity in minor cases, and in severe cases it may even cause complete crop failure. The disease may also impair the quality of the medicinal portions, lowering the therapeutic value of the plants. As a result, measures to minimize the negative impact of root rot on the production and quality of medicinal plants are essential to ensure the stability of the supply of medicinal herbs and to promote the sustainable use of medicinal plant resources.

## 6 Related factors of root rot occurrence in medicinal plants

**Figure 4:**
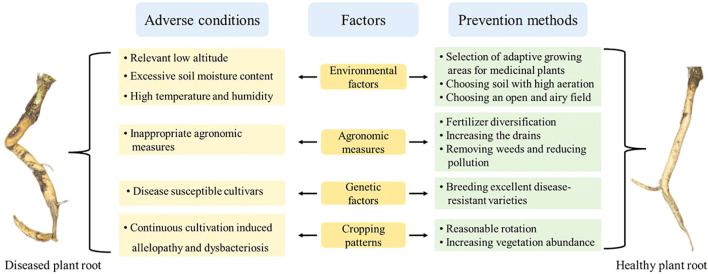
Factors associated with the occurrence of root rot in medicinal plants and prevention methods.

Root rot of medicinal plants primarily affects the roots and rhizomes of Chinese herbal medicines. Its typically triggered by a variety of pathogens that infiltrate through wounds in the plants roots or stems. Over the past decade, the incidence of root rot has become increasingly severe across various species of Chinese medicinal plants and in different herbal production regions nationwide, worsening year by year (Shen et al., 2014). Due to the factors that cause root rot are complex, no effective methods for prevention and control have yet been established. Therefore, identifying the causal factors associated with affected plants is crucial for effectively preventing and managing root rot. This part give priority to the influencing factors of root rot incidence in medicinal plants, examining aspects such as environmental conditions, genetic factors, field management, and plant continuous cropping (**Figure 4**).

### 6.1 Environmental factor

#### 6.1.1 Altitude

There is a correlation between the altitude of the area where medicinal plants are cultivated and the incidence of root rot. In Zhou’s study, the incidence of root rot in *P. notoginseng* was found to be 15.00% at altitudes below 500 meters. At altitudes ranging from 500 to 1000 meters, the incidence dropped to below 8.00%, and further decreased to 5.60% at altitudes between 1000 and 1300 meters (Zhou et al., 2016). The majority of the surveyed locations exhibited either sporadic occurrences of the disease or none at all, indicating a significant decline in root rot incidence of *P. notoginseng* with increasing altitude. Similarly, research on the incidence of root rot in *Aucklandia Lappa* at altitudes of 1250 to 1690 meters revealed a similar trend: root rot incidence decreased with rising altitude (Li et al., 2020). This phenomenon is primarily attributed to temperature variations. At higher altitudes, temperatures are relatively lower, which inhibits the growth of root rot pathogens. Conversely, as altitude decreases and temperatures rise, the warmer climate creates more favorable conditions for the growth and reproduction of these pathogens, leading to more severe disease occurrences.

#### 6.1.2 Soil moisture content

Soil plays a crucial role in influencing both plant health and adaptability, with soil moisture content playing a vital role in shaping the inter-root soil microbe of medicinal plants. The growth and development of plants and crops are inherently linked to water availability. Appropriate soil moisture is essential for the normal survival of plants. However, excessive water can lead to a higher incidence of plant diseases, jeopardizing plant survival. In the study by Zhao et al., the maximum water holding capacities (w) in the field were set at 45%, 60%, 70%, and 85% (Zhao et al., 2014). The results indicated a rapid increase in the incidence of root rot in *P. notoginseng* with rising soil moisture content. Specifically, the 85% w treatment resulted in a 166.67% increase in root rot incidence compared to the 70% w treatment. Yang et al. investigated the effect of different soil textures on the occurrence of root rot in *C. chinensis* (Yang L, et al., 2024). They analyzed 19 plots with varying soil textures and found that the root rot incidence in clayey soil was 15.51% higher than in loam soil. This rise in incidence can be linked to the enhanced water retention characteristic of clayey soils and low-lying regions. Under these conditions, the soil becomes severely oxygen-depleted, causing plant roots and rhizomes to shift from aerobic to anaerobic respiration. This anaerobic process generates significant amounts of ethanol, which can be toxic to the roots and rhizomes. Ethanol poisoning results in root and rhizome injuries, allowing pathogens to invade through these wounds, exacerbating rot and ultimately leading to root rot. Thus, it is evident that soil moisture levels profoundly influence the incidence of root rot.

#### 6.1.3 Temperature and humidity levels

The temperature and humidity of the environment significantly influences the occurrence, severity, and prevalence of root rot diseases in various medicinal plants. In China, areas such as Sichuan Province, which experience excessive rainfall, intensive irrigation, and poor soil drainage, have a higher incidence of oomycetes root rot compared to the northwest (Xinjiang and Gansu) and north (Inner Mongolia) regions. This results in a higher prevalence of root rot disease in *M. sativa.*

In the main production area of *P. notoginseng* in Wenshan, Yunnan, two peaks of disease occurrence are observed each year (Liang et al., 2006). The first peak occurs between March and April, during the period from sowing to seedling emergence, when relative humidity exceeds 85% (Cai et al., 2021). This primarily affects seedlings, often resulting in failure to emerge. The second peak appears from July to August, when relative humidity rises above 80%-90% and temperatures reach around 20 degrees Celsius, leading to rapid disease spread. These observations indicate that root rot is more likely to develop in cultivation areas characterized by high temperatures and high humidity.

### 6.2 Genetic factors

Root rots resistance varies significantly among individuals of different medicinal plants, and its occurrence is also linked to genetic factors of these plants. Chen et al. developed the first disease-resistant cultivar of *P. notoginseng*, named “Miaoxiang Kangqi 1” (Chen et al., 2017). Results from root rot resistance tests indicated that its two-year-old seedlings demonstrated substantial resistance to the root rot pathogen *F. oxysporum.* When compared to common cultivars, the disease index for root rot in these resistant varieties was notably reduced by 35.8% to 62.4%. By employing continuous breeding and promoting disease-resistant cultivars, root rot incidence was effectively managed at the genetic level, thereby minimizing the losses incurred by this disease in medicinal production.

### 6.3 Field management

Effective field management directly impacts the incidence of root rot. As a soil-borne ailment, root rot can be effectively mitigated by controlling the microbial population through management practices. For instance, burning diseased plant residues, stubble, and weeds can help reduce the spread of disease. Additionally, proper water and fertilizer management during cultivation significantly impacts root rot incidence. The use of green organic manure, farmyard manure, and organic fertilizers (such as compost, green manure, and animal manure) not only reduces the incidence of root rot but also fosters the growth of beneficial soil microorganisms (Wiggins and Kinkel, 2005). Wang et al. examined the incidence of root rot in *P. notoginseng* under various management practices, revealing a marked difference in disease severity between fine and loose management approaches (Wang et al., 1998). For instance, the incidence rate of root rot at Yanshan Farm, which is under careful management, was approximately 0.5%. In contrast, at Wenshan Gumu and Pingbazhai, where management practices were more lax, the incidence rate soared to 30.7%. This illustrates a significant disparity of 30% in the occurrence of diseased plants. Zhang et al. demonstrated that enhancing the application of compound fertilizers, phosphate fertilizers, and potash while maintaining local traditional fertilizer levels along with increasing the use of microbial fungicides, could reduce the incidence of *Pseudostellaria heterophylla* from 19.06% to 6.89% (Zhang et al., 2015). Similarly, Yao et al. managed to decrease the incidence of root rot in *A. membranaceus* from 52% to 34.1% by exclusively applying organic fertilizers instead of formulated chemical fertilizers. Furthermore, Yao et al. conducted a two-year study on the effects of different irrigation methods on root rot in *P. notoginseng* cultivation sites. Their results indicated that alternating irrigation volumes of 0.125 m³ and 0.375 m³ significantly reduced the occurrence of root rot (Yao et al., 2024). Overall, effective water and fertilizer management tailored to local conditions plays a crucial role in minimizing root rot incidence in plants.

### 6.4 Damage of continuous cultivation on root health of medicinal plants

Continuous cropping disorder refers to the phenomenon where planting the same or similar crops in the same soil for several consecutive years leads to growth retardation, quality deterioration, and yield reduction (Liu Z, et al., 2020). Yang et al. investigated the occurrence of root rot in four *S. miltiorrhiza* production areas in Henan Province, revealing average root rot rates of 7.2%, 34.2%, 32.2%, and 37.1%, respectively (Yang et al., 2021). Notably, the highest incidence of the disease was found in continuous cropping fields, where the rate of diseased plants reached 85.0%. In another study, Yang et al. selected 14 plots of *C. chinensis* with 3 to 4 years of growth to analyze root rot occurrence across different cropping systems (Yang L. et al., 2024). They found that the rates of root rot in *C. chinensis* were lower than 15.67% in barren plots and 20.97% in rotated plots, while continuous cropping plots exhibited a significantly higher root rot rate of 55.32%. Li et al. conducted research in Shibing County, Guizhou Province, where the incidence of root rot in *P. heterophylla* was relatively low at 2.00%. However, they discovered that the disease incidence and disease index for plots subjected to 2 years and 4 years of continuous cropping were 36.67% and 46.00%, respectively (Li et al., 2017). This indicates a strong correlation between the duration of continuous cropping and the occurrence of root rot disease in medicinal herbs.

Self-toxicity caused by allelopathic substances is recognized as a significant cause of root rot in medicinal plants, primarily due to the disorder associated with continuous cropping. Studies have demonstrated that, following prolonged continuous cropping, certain medicinal plants release harmful substances into the soil, which can directly or indirectly affect their own development and growth, leading to toxic effects known as chemosensory self-toxicity. Ren et al. indicated that changes in the content of β-ODAP are closely linked to the occurrence of root rot in *P. quinquefolius.* Specifically, the longer the period of continuous cropping, the greater the accumulation of β-ODAP and the more severe the incidence of root rot (Ren et al., 2018). It has been further hypothesized that the severity of root rot may be positively correlated with the concentration of β-ODAP secreted into the soil. Since over 70% of Chinese herbal medicines consist of roots and rhizomes, chemosensory substances in the soil can significantly disrupt the soil micro-ecosystem, causing severe damage to the growth of medicinal plants and consequently leading to a high incidence of root rot (Zhao et al., 2024).

Significant reductions in the number and diversity indices of inter-root fungi, along with notable changes in the community composition caused by continuous cropping, are widely recognized as the primary factors contributing to root rot in plants. Tan et al. demonstrated that the diversity of inter-root and endophytic fungi in *P. quinquefolius* soil significantly decreased as the number of years of continuous cropping increased (Tan et al., 2017). Yu et al. employed Illumina MiSeq high-throughput sequencing to analyze the changes in fungal community diversity and composition between unplanted *P. quinquefolius* soil and the inter-root soil of four-year-old healthy and root-rotted ginseng in a new stubble field (Yu et al., 2018). Their findings revealed a significant decline in both species richness and diversity indices of inter-root fungi in continuous four-year planting of *P. quinquefolius* compared to a blank field. Notably, the diversity index of soil fungi in the root-rotted ginseng was the lowest among the samples. Chen et al. investigated the bases of *P. quinquefolius* in the Huai-rou of Beijing, finding that the number of inter-root fungi in soil from heavily cropped *P. quinquefolius* decreased by 50% to 63%, while the diversity index (H’) dropped by 39% to 43% compared to new land where ginseng had not been planted (Chen et al., 2012). Lu et al. compared the inter-root soil fungal community structures between root-rot diseased and healthy *P. sibiricum*. Their results revealed that the number of operational taxonomic units in the soil samples from the diseased plant group was significantly lower than that in the healthy group and the blank group (Lu et al., 2021). The diversity index for the diseased group was the lowest, while the blank group exhibited the highest diversity. These findings underscore the multifaceted effects of plant succession on the health of medicinal plant root and highlight the need for strategic interventions to mitigate these challenges and sustain long-term productivity in medicinal plant cultivation.

## 7 Strategies for managing root rot in medicinal plants

**Figure 5:**
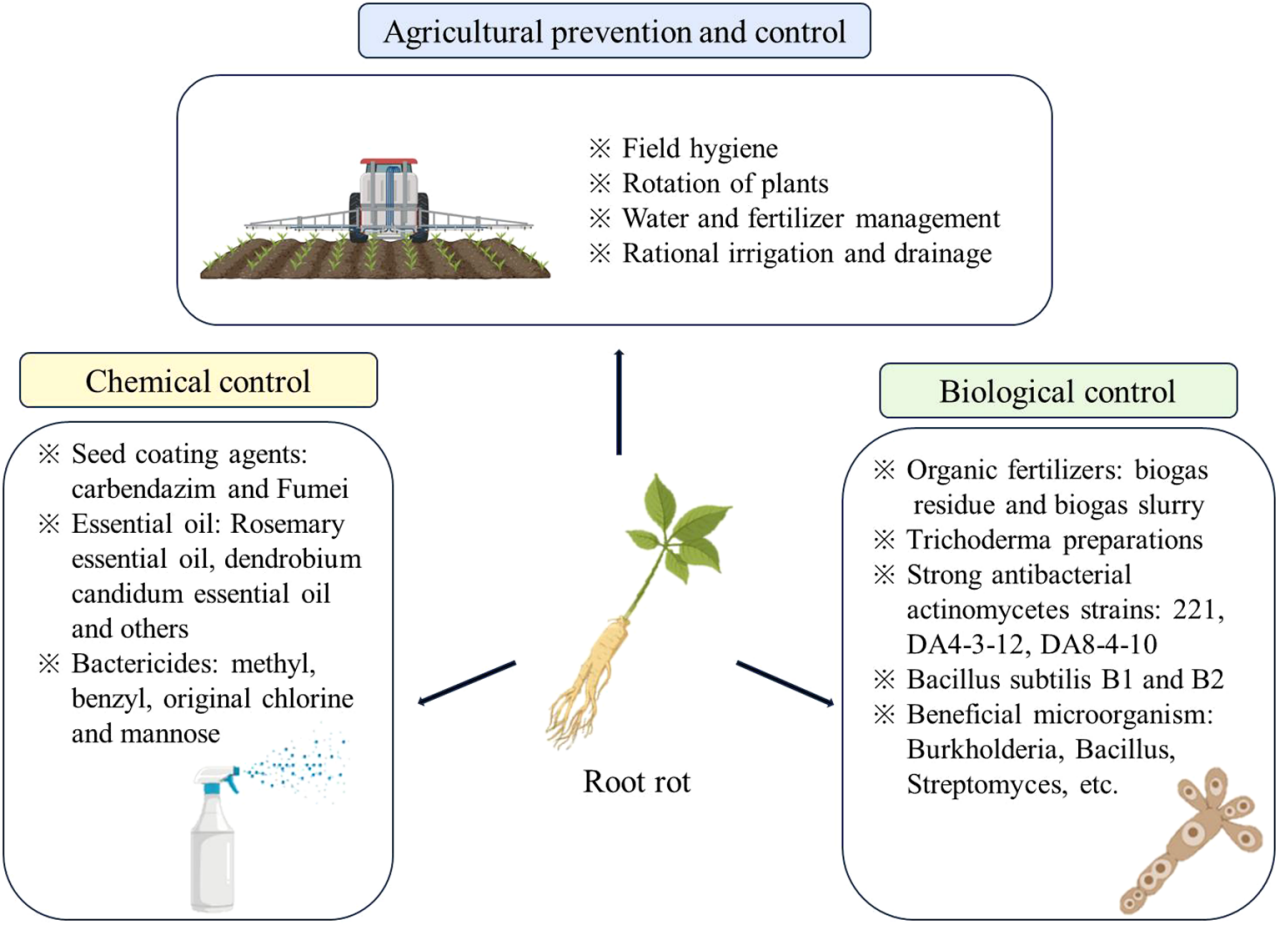
Control measures for root rot of medicinal plants. Images sourced from Scientific Image and Illustration Software | BioRender.

The escalating severity of root rot poses significant challenges to medicinal plants, prized for their therapeutic properties. This issue leads to reduced yield sand compromised quality, becoming a critical concern as global economic dynamics evolve with a heightened emphasis on quality standards. Successfully addressing root rot without heavily relying on chemical interventions has become a pressing challenge. Controlling soil-borne diseases, particularly root rot, is inherently complex. Contemporary approaches encompass a range of methods such as physical control, agricultural practices, chemical control, biological control, and integrated control strategies. These varied approaches aim to strike a careful balance, effectively managing root rot while promoting sustainable cultivation practices that minimize dependence on chemical inputs for medicinal plants (**Figure 5**).

### 7.1 Agricultural measures for prevention and control

Agricultural control is fundamental in combating root rot diseases. Select suitable planting plots and select high-quality seeds with high moisture content and purity for planting. The soil should be disinfected before planting to avoid mechanical damage to plants during planting. At the same time, careful water and nutrient management is implemented to ensure that the field has good drainage. These measures help to increase the yield of medicinal plants, improve nutrient absorption, and enhance the plant’s defense against external pathogens (Zhang J. N. et al., 2024; Yang Y. et al., 2024). Researchers have discovered that a balanced application of nitrogen, phosphorus, and potassium, coupled with the incorporation of green manure into the soil, can significantly reduce the incidence of root rot in plants of the Araliaceae family, achieving an impressive inhibition rate of up to 50% (Zhang et al., 2021). In another study, Liu and colleagues demonstrated that the use of bio-char to amend soils for continuous cropping not only boosts soil fertility, but also diminishes the relative abundance of root rot pathogens (Liu C. et al., 2022).

Exquisite agricultural field management, such as the adoption of rational crop rotation and intercropping, can use the chemical sensitive substances secreted by the roots of different plants to overcome the barriers of continuous cropping (Chen et al., 2022). Reductive soil disinfestation is a method of soil treatment before planting crops, which can be used as a barrier removal technique to rebalance the soil microflora of *P. ginseng* cultivation (Li et al., 2019; Duan et al., 2025). Native microbiome inoculation restore soil capacity to suppress a root disease (Zhou et al., 2023). Studies have shown that the soil under continuous cropping has higher fungal diversity, the organic matter, pH, catalase and other enzyme activities in the soil are significantly reduced, while the content of available phosphorus (AP) and potassium (AK) are significantly increased. Crop rotation is a very important means, which can change the structure of soil microbial communities, affecting the ecological function of the soil. Liu found that rotating corn or cowpea with ginseng can reduce the root rot of ginseng, corn rotation can increase root biomass and reduce root rot, significantly increasing the relative abundance of beneficial bacteria (Liu C. et al., 2022). The impact of different rotational crops on microbes varies, making the selection of suitable rotational plants very important. The advantage of agricultural management lies in its environmental friendliness, avoiding negative effects on soil and plants, but it requires long-term experience and practice, and its impact will not be particularly significant.

### 7.2 Chemical control

Chemical control, characterized by its swift action, simplicity, and immediate results, serves as a crucial tactic in the management of medicinal plants. According to the statistics of Pesticide Information Network (http://www.jsppa.com.cn/news/yanfa/11912.html), a total of 286 pesticides are registered for root rot control in China, of which the most registered is fludioxonil, followed by propiconazole, thiram, hymexazol and so on. Researchers including Ma utilized carbendazim and Fumeishuang as seed treatments to inhibit the pathogens causing root rot in legumes (Ma et al., 2010). Similarly, Li and colleagues found that the application of 98% dazomet granules, chloropicrin, and phenyl compounds to soils in continuously cropped areas significantly suppressed root rot in Araliaceae plants (Li Z. et al., 2022). The triazole compound agent benzoyl propiconazole has the best control effect on *F. oxysporum*, mancozeb and fludioxonil can produce different degrees of inhibition on a variety of *Fusarium* fungi (Ma et al., 2022; Zhang T. et al., 2024). Pyraclostro-bin, metconazole, saphire and tebuconazole exhibited relative higher inhibitory on *F. proliferatum* (Wu et al., 2023). Pentazolol, fluzoyl hydroxyl amine, pyrimethystrobin and thiopazim can be used as rotating agents to slow the development of resistance in pathogenic bacteria (Cui et al., 2022).

Despite its efficacy, chemical control faces challenges in the production of Chinese medicinal materials, which is predominantly managed by individual farmers. These farmers often lack professional knowledge, leading to excessive use of potent, low-cost pesticides (Duke, 2018). This practice not only results in the violation of pesticide residue standards, but also compromises the quality of medicinal materials. It has sparked concerns over environmental contamination and the rise of pathogen resistance to treatments, thereby restricting the broad adoption of chemical control methods (Tudi et al., 2021).

### 7.3 Biological control

Biological control methods mainly utilize organic fertilizers to modulate the bacteria associated with plant roots. Some studies evidence the preventative and curative effects of biogas residue and biogas liquid on root rot disease. Fungi and mycoviruses have biocontrol capabilities to resisting pests like nematodes and microbial pathogens that infect various parts of the plant, such as yeasts, *Trichoderma harzianum* and *Bacillus subtilis*. These are biocontrol agents that have been effectively commercialized (Seitz et al., 2023; Umer et al., 2023; Freimoser et al., 2019; Wang Y. et al., 2023). *T. harzianum* controls rates of root rot over 70%, increased yield enhancements between 14.2% and 24.5% for *L. chuanxiong*, *S. miltiorrhiza*, and *O. japonicus.* It also have antagonistic effects against root rot in leguminous plants and also promote plant growth. *B. subtilis* and *B. pumilus* have shown notable efficacy in the control of root rot for Asteraceae and Fabaceae plants, achieving inhibition rates of 72.79% to 81.09% (Bae et al., 2011; Park et al., 2019; Li et al., 2007). Streptomyces *XTBG45* can enrich the healthy rhizosphere and effectively control root rot caused by *F. oxysporum* and *anthracnose* (Pang et al., 2022). *Arbuscular mycorrhizal* as they have been shown to reduce the incidence of fungal diseases and nematode attacks on host plants by 30%-42% and 44-57%, respectively (Basiru et al., 2023). Besides, microbial volatile compounds, like *Bacillus* species, that can be used for biocontrol of plant pathogens, such as bacteria, oomycetes and fungi, are a sustainable first choice for synthetic fungicides (Xia et al., 2023; Tilocca et al., 2020).

Phage cocktails are a feasible option for controlling a variety of plant diseases (Kering et al., 2019; Mani, 2023). We can also insert mutations into specific regions in the genome with high precision, and efficiency using techniques like Crispr/Cas and mutations. It can be induced in numerous genes at the same time, which aid in determining the role of different genes in biocontrol (Park et al., 2024). Microbiome engineering is an intriguing option for improving a plant’s biological capabilities, which has the potential to have a big impact on agriculture (Afridi et al., 2022; Song et al., 2023; Jansson et al., 2023). Predatory protists may therefore represent promising biological agents that can contribute to sustainable agricultural practices by promoting interactions between the plant and its microbiome (Guo et al., 2024). In addition, Agricultural Jiaosu has effectively managed *F. oxysporum* with a semi-maximum inhibitory concentration of 13.64% (Gao H, et al., 2022). Biological control methods are lauded for their minimal environmental impact, their capacity to sustain the equilibrium of soil microbial communities, and their role in maintaining the stability of agricultural ecosystems. Nevertheless, these approaches are highly susceptible to variations in field environments and weather conditions, which can restrict their practical application in farming (Gao et al., 2022; Ma et al., 2021; Tsai et al., 2023).

## 8 Genetic mechanism in the occurrence and control of root rot

As the demand for medicinal plants continues to grow, the cultivation area of medicinal plants with roots and root tubers as the primary medicinal parts is also expanding. However, the high incidence of root rot disease is one of the main reasons for the reduction in the yield of medicinal plants. While it is currently recognized that pathogens are the major cause of root rot in medicinal plants, efforts to reduce the occurrence of this disease through optimized management practices and the application of chemical pesticides during cultivation and management have not yielded satisfactory results. Therefore, there is an urgent need for the cultivation of new medicinal plant varieties that are highly resistant to disease, the mechanisms of disease resistance is a prerequisite for breeding highly resistant varieties of medicinal plants.

Throughout the process of plant growth and evolutionary development, plants are often threatened by various pathogenic microorganisms. In addition to producing a range of secondary metabolites as a result of evolution, they also generate a series of immune regulatory responses. During the plant immune response, resistance genes play a crucial role in defending against pathogen invasion. To date, more than 100 disease-resistant genes have been cloned from various plants (Dangl and Jones, 2001), such as soybean (Niu et al., 2022; Million et al., 2019; Gao et al., 2024; You et al., 2023; Gao Y. et al., 2022; Guo et al., 2022; Shao YY, et al., 2024), wheat (Geng et al., 2016; Wang N. et al., 2022; Wang et al., 2014), maize (Li et al., 2023), rice (Tian et al., 2022; Wang N et al., 2022; Tao et al., 2021), tobacco (Gai et al., 2021), pea (Kälin et al., 2024), *Lilium regale* (Li S. et al., 2024), apple (Pei et al., 2024), and *Cicer arietinum* (Sadhu et al., 2023) (**Table 2**). The research on the genetic mechanism of root rot disease in medicinal plants started later than that in crops, but the discovery and functional verification of disease-resistant genes are also developing rapidly (Jamann et al., 2016; Wang H. L. et al., 2024; Pépin et al., 2021; Wang et al., 2014; Ding et al., 2023; Parkunan et al., 2014; Shao W. et al., 2024). In root rot research on medicinal plants, the use of transcriptome and resequencing technologies to screen for resistance-related regulatory genes against root rot has emerged as a new approach for developing root rot-resistant varieties. Kang et al. screened for disease resistance genes through transcriptomic analysis of the roots of *P. notoginseng* and conducted quantitative analysis of hormone levels using UPLC-MS. The results indicated that the transient overexpression of the gene *PnWRKY22* increased salicylic acid levels in the leaves of Sanqi, enhancing its resistance to root rot disease, suggesting that *PnWRKY22* is an important disease resistance gene for *P. notoginseng* root rot (Kang et al., 2021; Ning et al., 2021; Li S. et al., 2021). Li et al. showed that the *PnPR-like* gene exhibited significant *in vitro* antifungal activity, and the overexpression of *PnPR-like* resulted in transgenic tobacco plants displaying high resistance to the root rot pathogen *F. solani* (Li et al., 2020). Zheng et al. demonstrated that the transcription factor *PnWRKY9* could activate the transcription of the defense-related defensin gene *PnDEFL1* against *F. solani*, and that *PnWRKY9* works synergistically with the jasmonic acid (JA) signaling pathway to enhance disease resistance, indicating that *PnWRKY9* has a positive effect on plant defenses against root rot pathogens (Zheng et al., 2022). Zhao et al. conducted transcriptomic analysis on two disease-resistant genotypes of the same variety of M. sativa that showed different resistance levels to root rot, successfully screening for the candidate disease resistance gene *MsSPL15* (Zhao et al., 2023). By investigating the resistance and susceptibility genes associated with root rot disease, we can gain deeper insights into the mechanisms of disease resistance in medicinal plants. This research provides a foundation for tackling the challenges posed by root rot disease and for the development of disease-resistant varieties of medicinal plants (Gao Y. et al., 2022; Li et al., 2024; Zhang X. et al., 2024; Song S. et al., 2023; Sun B. et al., 2024).

**Table 2:** Disease resistance related genes.

Source plants		Genes	Diseases	Bio-function
Medicinal plants	*P. notoginseng*	*PnWRKY22*	Root rot	Resistant gene
	*P. notoginseng*	*PnPR-like* gene	Root rot	Resistant genes
	*P. notoginseng*	*PnWRKY9*	Root rot	Resistant gene
	*P. notoginseng*	*PnCHI3*	Root rot	Resistant gene
	*M. sativa*	*MsSPL15*	Root rot	Resistant gene
	*C. sativa*	*CsMLO1、CsMLO4*	Powdery mildew	Susceptible genes
	Chrysanthemum	*CmbHLH18*	Black spot disease	Resistant gene
Crops and other plants	Tobacco	*NtSWEET1*	Root rot	Resistant gene
	Pea	*Psat7g091800.1*	Root rot	Resistant gene
	*L. regale*	*LrWRKY2*	Root rot	Resistant gene
	Apple	*MdCERK1*	Root rot	Susceptible gene
	Soybean	*GmMYB78*	Phytophthora root rot	Susceptible gene
	Soybean	*RpsSDB*	Phytophthora root and stem rot	Resistant gene
	Soybean	*GmMPK6*	Phytophthora root and stem rot	Resistant gene
	Soybean	*CaAMP1*	Phytophthora root and stem rot	Resistant gene
	Soybean	*TaBln1*	Stripe rust	Susceptible gene
	Soybean	*SRA2、SRZ4*	Soybean Mosaic Virus、Tobacco mosaic virus	Resistant genes
	Wheat	*TaWRKY19*	Stripe Rust	Susceptible gene
	Wheat	*Rps14*	Phytophthora sojae	Resistant gene
	Wheat	*Mlo* locus	Powdery mildew	Susceptible genes
	Wheat	*MLIW30*	Powdery mildew	Resistant gene
	Maize	*ZmNANMT*	Southern leaf blight、northern leaf blight、*Fusarium* stalk rot	Resistant gene
	Rice	*Pi21、Bsrd1、Xa5*	Rice blast、bacterial blight	Resistant genes
	*C. arietinum*	*RsAFP2*	*Fusarium* wilt	Resistant gene
Source plants		Genes	Diseases	Bio-function
Medicinal plants	*P. notoginseng*	*PnWRKY22*	Root rot	Resistant gene
	*P. notoginseng*	*PnPR-like* gene	Root rot	Resistant genes
	*P. notoginseng*	*PnWRKY9*	Root rot	Resistant gene
	*P. notoginseng*	*PnCHI3*	Root rot	Resistant gene
	*M. sativa*	*MsSPL15*	Root rot	Resistant gene
	*C. sativa*	*CsMLO1、CsMLO4*	Powdery mildew	Susceptible genes
	Chrysanthemum	*CmbHLH18*	Black spot disease	Resistant gene
Crops and other plants	Tobacco	*NtSWEET1*	Root rot	Resistant gene
	Pea	*Psat7g091800.1*	Root rot	Resistant gene
	*L. regale*	*LrWRKY2*	Root rot	Resistant gene
	Apple	*MdCERK1*	Root rot	Susceptible gene
	Soybean	*GmMYB78*	Phytophthora root rot	Susceptible gene
	Soybean	*RpsSDB*	Phytophthora root and stem rot	Resistant gene
	Soybean	*GmMPK6*	Phytophthora root and stem rot	Resistant gene
	Soybean	*CaAMP1*	Phytophthora root and stem rot	Resistant gene
	Soybean	*TaBln1*	Stripe rust	Susceptible gene
	Soybean	*SRA2、SRZ4*	Soybean Mosaic Virus、Tobacco mosaic virus	Resistant genes
	Wheat	*TaWRKY19*	Stripe Rust	Susceptible gene
	Wheat	*Rps14*	Phytophthora sojae	Resistant gene
	Wheat	*Mlo* locus	Powdery mildew	Susceptible genes
	Wheat	*MLIW30*	Powdery mildew	Resistant gene
	Maize	*ZmNANMT*	Southern leaf blight、northern leaf blight、*Fusarium* stalk rot	Resistant gene
	Rice	*Pi21、Bsrd1、Xa5*	Rice blast、bacterial blight	Resistant genes
	*C. arietinum*	*RsAFP2*	*Fusarium* wilt	Resistant gene
Source plants		Genes	Diseases	Bio-function

## 9 Discussion

To date, research on root rot in medicinal plants has primarily focused on the families such as Araliaceae, Fabaceae, Ranunculaceae, and Solanaceae. Given the growing concern regarding root rot in medicinal plants, this review aims to present the current state of research in this field. By summarizing the progress made in root rot of medicinal plants, it seeks to provide valuable references for related studies, including effective disease prediction, control measures, and the standardized cultivation of medicinal plants. It also proposes reasonable future research directions in the area of root rot in medicinal plants.

The prevention and control of root rot in medicinal plants is a valuable research field. Although existing studies have achieved diversified progress in the research and prevention of root rot, mainstream control methods, including agricultural management, biological control, and chemical control, exhibit their own limitations. Although agricultural and biological controls are environmentally friendly, they tend to be slow-acting and may not be effectively applied during the mid-to late stages of root rot outbreaks. On the other hand, while chemical control is highly efficient, it presents challenges such as drug residues and the gradual development of pathogen resistance. Furthermore, significant differences among plant types, the complexity of pathogenic causes, poor varietal stability, and long breeding cycles have impeded the progress of selecting disease-resistant germplasm. Thus, there is an urgent need to devise reasonable methods for effectively preventing root rot in medicinal plants to guide scientifically sound cultivation practices. In terms of biological control, optimizing the formulation of biological fertilizers and other biocontrol agents can enhance the efficacy of biological control while achieving a balance between environmental safety and practical efficiency. In plant breeding, in addition to traditional germplasm selection methods, utilizing multi-omics technologies to identify disease-resistant genes can speed up the breeding process. Genome editing technologies for plants offer vast opportunities for the development of disease-resistant varieties in medicinal plants. The ability to achieve efficient, precise, and targeted mutations throughout the entire genome, combined with complementary technologies such as high-throughput phenotyping, genomic selection, and rapid breeding, holds promise for ensuring the widespread application of genome editing.

In addition to precise control, effective prediction is also crucial for mitigating disease severity and reducing losses. As a typical soil-borne disease, root rot initially manifests in the roots and root tubers of plants before symptoms appear on the entire plant. This often results in growers being unable to monitor and control the disease in its early stages. Therefore, the development of reliable and non-destructive early disease detection technology is vital. For example, hyperspectral reflectance imaging technology was used for non-destructive detection of carotenoids, starch, and sucrose levels in *P. ginseng*, enabling real-time monitoring and prediction of root rot occurrence based on detection results (Park et al., 2023). Similarly, hyperspectral imaging technology was employed to detect spectral changes in chili leaves to predict root rot incidence (Shao YY, et al., 2024). However, due to significant individual variation among medicinal plants, detection technologies that are applicable to a single plant species may have limited applicability. Therefore, there is an urgent need to develop early detection techniques that can be broadly applied to root rot across various plant species.

The occurrence of root rot in medicinal plants is the result of a complex interplay between pathogens and environmental conditions. The onset of root rot is closely related to the environmental factors affecting the growth of medicinal plants, field management practices, and individual variations among plants. With global climate change and shifts in agricultural production patterns, the incidence of root rot may also change. Future research should focus more on the dynamic monitoring of root rot under different environmental and management conditions, as well as its interactions with the growth of medicinal plants. By deepening our understanding of the mechanisms underlying root rot occurrence and its influencing factors, we can integrate modern biotechnology with traditional agricultural management practices to promote the breeding of quality varieties and sustainable cultivation of medicinal plants, thereby ensuring the healthy development of the medicinal plant industry, which is also critical for maintaining the economic viability of medicinal plant cultivation.
